# Effect of vitamin D supplementation on CREB-TrkB-BDNF pathway in the hippocampus of diabetic rats

**DOI:** 10.22038/IJBMS.2019.38170.9068

**Published:** 2020-01

**Authors:** Hoda Nadimi, Abolghassem Djazayery, Mohammad Hassan Javanbakht, Ahmadreza Dehpour, Ehsan Ghaedi, Hoda Derakhshanian, Hamed Mohammadi, Seyedeh Neda Mousavi, Mahmoud Djalali

**Affiliations:** 1 Department of Cellular and Molecular Nutrition, School of Nutritional Sciences and Dietetics, Tehran University of Medical Sciences, Tehran, Iran; 2 Department of Community Nutrition, School of Nutritional Sciences and Dietetics, Tehran University of Medical Sciences, Tehran, Iran; 3 Experimental Medicine Research Center, Tehran University of Medical Sciences, Tehran, Iran; 4 Students’ Scientific Research Center (SSRC), Tehran University of Medical Sciences (TUMS), Tehran, Iran; 5 Department of Biochemistry, Nutrition and Genetics, Medical School, Alborz University of Medical Sciences, Karaj, Iran; 6 Dietary supplements and probiotics Research Center, Alborz University of Medical Sciences, Karaj, Iran; 7 Student Research Committee, Department of Clinical Nutrition, School of Nutrition and Food Science, Isfahan University of Medical Sciences, Isfahan, Iran; 8 Department of Biochemistry and Nutrition, School of Medicine, Zanjan University of Medical Sciences, Zanjan, Iran

**Keywords:** Brain-derived neurotrophic- factor, CREB, Diabetes, TrkB, Vitamin D

## Abstract

**Objective(s):**

Cyclic AMP (adenosine monophosphate) response element-binding protein (CREB) and Brain-derived neurotrophic factor (BDNF) are reported to broadly involve in learning capacity and memory. BDNF exerts its functions via tropomyosin receptor kinase B (TrkB). BDNF transcription is regulated by stimulating CREB phosphorylation. The CREB-TrkB-BDNF pathway is reported to be affected by diabetes, which may contribute to its cognitive deficits. This study was conducted to investigate the effect of vitamin D supplementation on the hippocampal fraction of this pathway in an animal model of type-1 diabetes mellitus (T1DM).

**Materials and Methods:**

Thirty-six adult male Sprague-Dawley rats were randomly divided into 4 groups as follows: Group 1: normal healthy rats (n=8); group 2: normal healthy rats receiving sesame oil supplementation as placebo (n=8); Group 3: diabetic rats receiving sesame oil (n=10); and Group 4: diabetic rats treated with 4300 IU/kg/week vitamin D dissolved in sesame oil (n=10). Diabetes was induced by intraperitoneal (IP) injection of streptozotocin. Blood and hippocampal samples were acquired at the end of the experiment. RNA was extracted from the hippocampus, and real-time PCR (polymerase chain reaction) was performed for BDNF and TrkB gene expression.

**Results:**

Administration of vitamin D (4300 IU/kg/week) in a T1DM animal model increased CREB phosphorylation in the hippocampus, but the serum and hippocampal BDNF levels and TrkB and BDNF gene expression did not change significantly.

**Conclusion:**

Vitamin D increased hippocampal CREB phosphorylation in a T1DM animal model. Our findings showed that vitamin D might be protective against central nervous system complications in diabetes. However, future studies are warranted.

## Introduction

Diabetes mellitus (DM) is a multi-systemic chronic disorder characterized by persistent high blood glucose levels. In fact, insulin cannot perform its main roles because of defects in insulin secretion, action, or both (1). The prevalence of DM as one of the most prevalent endocrine disorders is rising all over the world (2). Based on the World Health Organization reports, the global prevalence of DM will reach more than 3.6% by 2030 (3). Long-term hyperglycemia could lead to several chronic end-organ complications in the eyes, kidneys, and brain (4, 5). Diabetes and chronic hyperglycemia may result in central nervous system (CNS) complications. The exact mechanism causing cortical and cerebellar dysfunction is not elucidated yet, but brain cells are reported to be vulnerable to oxidative stress (6). Prolonged hyperglycemia in a pro**-**inflammatory setting disturbs the normal oxidant-antioxidant balance, resulting in oxidative stress and injury in diabetes (7, 8). 

Cyclic AMP (adenosine monophosphate) response element-binding protein (CREB) is reported to widely involve in learning capacity and memory (9). Its activated form, phosphorylated CREB (pCREB), plays significant roles in hippocampal neurogenesis, neuroprotection, and neuronal differentiation (9). Brain-derived neurotrophic factor (BDNF) is a major neurotrophic factor synthesized by neurons in the frontal cortex and hippocampus of rodents (10). BDNF is also involved in neuronal development, synaptogenesis, learning, and memory (9). It supports the survival of cholinergic and dopaminergic neurons (10). BDNF exerts its functions via activation of a specific receptor, i.e., tropomyosin receptor kinase B (TrkB) (11). BDNF transcription is regulated by stimulating CREB phosphorylation (12). BDNF, TrkB, and its downstream target CREB are reported to be involved in depression, as well (13). Both BDNF and CREB, through anti-apoptotic family members, lead to neuronal survival (14)*.* Several studies found that oxidative stress decreases antioxidant enzymes and BDNF levels and impairs the mitochondrial function (12). CREB expression is also decreased following an increase in ROS and other inflammatory insults. In diabetes, hyperglycemia, advanced glycation end-products (AGE), lipids, and chemokines all increase the intracellular oxidant load (15). CREB has been recognized as an important transcriptional factor for preserving an effective glucose sensing, insulin secretion, insulin gene transcription, and β-cell survival. CREB activates the transcription of target genes in β-cells in response to different stimuli, including glucose, incretin hormones, and growth factors. These activation mechanisms, including a diverse spectrum of protein kinases and their cofactors, allow CREB to modulate the expression of crucial genes such as insulin or insulin receptor substrate (16). On the other hand, decreased pCREB is identified as one of the main reasons for cognitive decline in diabetes, maybe through decreased insulin levels (17, 18). Several studies have investigated the association between BDNF and diabetes in experimental and clinical diabetes (19). Low BDNF levels are reported in diabetic humans and mice (19). According to published reports, it seems that diabetes decreases BDNF levels by increasing oxidative stress or by other independent mechanisms of oxidative stress. Therefore, restoration of the redox state in diabetes may restore low BDNF levels, which can prevent complications associated with diabetes and the development of neurodegenerative diseases (19).

Vitamin D (cholecalciferol) is a fat-soluble vitamin with a well-known role in calcium metabolism as well as insulin secretion and synthesis (20). Vitamin D receptors (VDR) are identified in different areas of the brain, like the prefrontal cortex, hippocampus, thalamus, and hypothalamus. Vitamin D3 may protect the structure and integrity of the neurons through detoxification pathways and neurotrophin synthesis (21)*. *Besides its antioxidant effects, increased BDNF and CREB expression levels are reported in experimental studies of the brain function (21, 22). 

This study was conducted to investigate the possible neuroprotective effects of vitamin D in an animal model of type-1 DM with distinct concerns regarding its possible mechanism of action through the CREB-trkB-BDNF pathway in the hippocampus. 

## Materials and Methods

***Experimental animals***

Thirty-six adult male Sprague–Dawley rats weighing 150–250 g were purchased from the Central Animal House, School of Pharmacology, Tehran University of Medical Sciences (TUMS), Tehran, Iran. All experimental animals were kept in standard cages under approved conditions, including an environmental temperature of 22±2 ^°^C and humidity of 55–65% with a 12-hr light-dark cycle. All animals had *ad libitum* access to a usual animal diet (Pars Dam Co, Tehran, Iran) and tap water. The animals were housed in separate cages in groups of four. All experimental procedures were in accordance with the standards of animal care approved by the Institutional Animal Care and Use Committee of TUMS (ethics code 28826/103/01/94). 

***Induction of type-1 diabetes model***

In the present study, type-1 diabetes was induced by an intraperitoneal (IP) injection of streptozotocin (STZ) (50 mg/kg) (Sigma-Aldrich Co. St Louis, MO, USA). The rats were fasting overnight before induction, and a single-dose of STZ dissolved in 0.1 M cold sterile sodium citrate buffer (PH: 4.5) was injected to all animals except the control rats. The development of T1DM was evaluated by fasting blood sugar (FBS) measurements. For this purpose, blood samples obtained from the tail veins were tested for FBS 72 hr after STZ injection by a glucometer (Bionime GM300, Swiss Design, Berneck, Switzerland). FBS levels of more than 250 mg/dl were considered diabetic. Other animals with no diabetes induction were excluded from the following experiments. Rats in the healthy control group received 1 ml sterile citrate buffer alone through an IP injection.

***Study design***

After two weeks of adaptation, the rats were divided into the following groups using a block randomization method: 

Group I (n= 8): Normal Control (NC) rats (1 ml sodium citrate through IP injection)

Group II (n= 8): NC rats receiving sesame oil (SO) (1 ml sodium citrate and 0.5 ml sesame oil through IP injection)

Group III (n= 10) : DM rats treated with SO (0.5 ml sesame oil through IP injection)

Group 4 (n= 10): DM rats treated with vitamin D (4300 IU/rat/week vitamin D (Osveh Co., Iran) dissolved in 0.5 ml sesame oil through IP injection)

All groups were treated with sesame oil or vitamin D for 4 weeks. In diabetic groups, two rats died during the study period; therefore, the analysis was performed for 32 rats. Twenty hours before the last food intake, the animals were kept fasting overnight. All rats were sacrificed after anesthetization by IP injection of ketamine (50 mg/kg) and xylazine (30 mg/kg). Blood samples were collected by heart puncture. The samples were promptly transferred to centrifuge tubes and centrifuged at 3500 rpm for 20 min. Serum samples were kept at -70 ^°^C until biochemical analysis. Bodyweight and food intake were recorded weekly during the experiment. 

***Hippocampus sample preparation***

Hippocampus samples were taken from all rats for measuring the total protein of phosphorylated CREB, BDNF, and trkB messenger ribonucleic acid (mRNA) expression. Fifty milligrams of hippocampus samples were diluted in 10 ng phosphate-buffered saline (PBS) (PH=7.4) and then homogenized by a homogenizer. The samples were centrifuged at 2000–3000 RPM for 20 min and the supernatants were collected carefully for measuring the total protein of phosphorylated CREB, BDNF, and trkB mRNA expression. All samples were kept at -80 ^°^C. 

***Biochemical measurements:***

FBS was measured by an enzymatic method and glucose oxidase method (Pars Azmoon kit, Iran). Serum vitamin D was measured using a commercial ELISA kit (Immunodiagnosticsystems (IDS) CO, London,UK) and expressed as ng/ml. Hemoglobin A1c (HbA1C) was measured before serum separation by an auto-analyzer using a commercial kit (BT-1500, Biotecnica Instruments, Italy). The circulating insulin level was evaluated by an ELISA kit (Mercodia Corporation, Uppsala, Sweden). This kit had an assay sensitivity of 1 mIU/ml. Intra-assay and inter-assay coefficient of variation (CV) were 2.1% and 6.5%, respectively. The total protein of phosphorylated CREB in the hippocampus was measured by an ELISA kit (Mercodia, Uppsala, Sweden). Serum BDNF was also measured by the ELISA method (Mercodia, Uppsala, Sweden), and the results are presented as ng/ml. 

***RNA extraction and real-time PCR gene expression quantification***

The extraction of cytoplasmic RNA from prepared tissue samples was performed using the RiboEx isolation kit (QIAGEN, Lilden, Germany) according to the manufacturer’s protocol. The sequences of primers used for real-time PCR reactions are presented in Table 1. The quality and the quantity of the extracted RNA were evaluated by a NanoDrop spectrophotometer (Thermo Fisher Scientific, San Jose, CA, USA). cDNA was synthesized using the QuantiTect Reverse Transcription Kit (Takara-Clontech, Tokyo, Japan). Quantitative real-time PCR was performed using the SYBR Premix Ex Taq II (Takara-Clontech, Tokyo, Japan). The gene expression changes compared to the housekeeping glyceraldehyde 3-phosphate dehydrogenase (GAPDH) gene were measured by the 2^ΔCt^ method (23).

***Statistical analysis***

The data are expressed as mean±standard deviation (SD). The Kolmogorov-Smirnov test was applied to check the normality of the data. One-way analysis of covariance (ANCOVA) followed by the Bonferroni *post hoc* test was used for data analysis. All analyses were adjusted for weight and food intake. *P*-values less than 0.05 were considered significant. SPSS version 17.01 (SPSS Inc., Chicago, USA) was used for statistical analysis.

## Results

***Effects of vitamin D supplementation on body weight, food intake, glycemic indices, and serum vitamin D level***

After induction of diabetes, body weight decreased in the diabetic control group compared to the healthy control group (*P*-value>0.05) (Table 2). However, vitamin D supplementation in T1DM-induced rats did not change body weight significantly compared to diabetic controls; however, the weight of the DM-Vit D group remained significantly lower than the healthy control group (*P*-value<0.05).

Table 2 shows the food intake, FBS, HbA1c, glycated albumin, serum insulin, and serum vitamin D of all experimental groups before and after intervention. Induction of diabetes led to a significant increase in FBS and HbA1C compared to the healthy control group (*P*-value<0.05). After the intervention, the results showed that administration of vitamin D did not change the FBS level compared to the DM group; however, HbA1C decreased significantly in comparison with the DM group. T1DM induction also decreased the serum insulin level in the DM group compared to the control group. Vitamin D administration did not change serum insulin in diabetic mice compared to the DM control group. Glycated albumin did not change significantly among experimental groups (*P*-value>0.05). Food intake was not significantly different between experimental groups at the beginning of the study as well (*P*-value >0.05). Induction of diabetes significantly increased food intake in the DM group, which did not change after administration of vitamin D; however, food intake was significantly higher in the DM group compared to the control group (*P*-value<0.05). Therefore, vitamin D supplementation in diabetic rats did not affect food intake significantly. As anticipated, administration of vitamin D in diabetic rats increased the serum level of vitamin D significantly compared to healthy and diabetic control groups (*P*-value<0.05). 

***Effects of vitamin D administration on serum BDNF***

As outlined in Table 2, induction of diabetes using STZ did not change serum BDNF significantly. Furthermore, administration of vitamin D for 4 weeks did not significantly change this variable in diabetic rats (*P*-value>0.05). 

***Effects of vitamin D administration on hippocampus total protein of phosphorylated CREB and BDNF***

**Figure 1:**
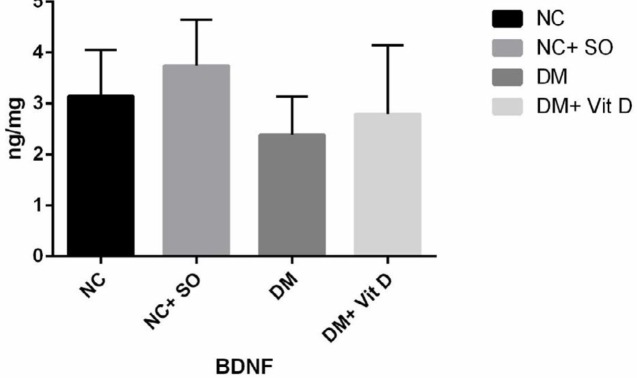
Total hippocampal BDNF protein in different experimental groups of rats. Results are expressed as mean±SD, one-way ANOVA, and *post hoc *Bonferroni test. There were no significant differences between groups. DM: diabetes mellitus, NC: normal control; SO: sesame oil; BDNF: brain-derived neurotrophic factor

**Figure 2:**
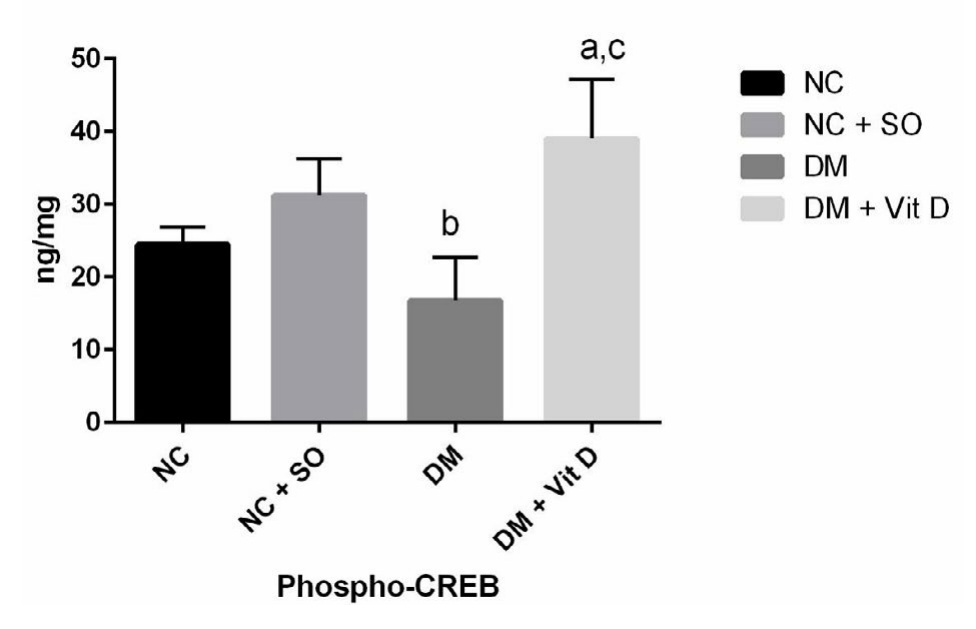
Hippocampal phosphorylated CREB in different experimental groups of rats. Results are expressed as mean±SD, one-way ANOVA, and *post hoc* Bonferroni test. DM: diabetes mellitus, NC: normal control, SO: sesame oil; CREB: cAMP-response-element binding protein a *P*<0.05 compared with the control group, b *P*<0.05 compared with the control group with sesame oil. c *P*<0.05 compared with the diabetic control group

Total hippocampal BDNF protein did not change after induction of diabetes (2.38±0.75 ng/mg), and differences were not significant compared to the healthy control group (3.14±0.91 ng/mg). Administration of vitamin D after induction of diabetes also did not change total hippocampal BDNF protein (2.79±1.35 ng/mg) (*P*-value >0.05) (Figure 1). However, induction of diabetes significantly decreased hippocampal phosphorylated CREB. In addition, administration of vitamin D in diabetic rats significantly increased hippocampal phosphorylated CREB in VitD + DM group compared to the diabetic (DM) and healthy control groups (*P*-value <0.05) (Figure 2).

***Effects of vitamin D supplementation on BDNF and TrkB gene expression in hippocampus tissue***

Real-time polymerase chain reaction (PCR) revealed that TrkB gene expression did not change in the hippocampal tissue of DM rats after induction of DM compared to the normal control group (Table 3). Administration of vitamin D after DM induction did not lead to significant changes in TrkB gene expression compared to the diabetic control group, either. In addition, there were no significant differences in the TrkB gene expression compared to GAPDH between DM + Vit D and healthy control groups. BDNF gene expression in the hippocampus tissue did not change after DM induction in the DM group, either. Vitamin D supplementation in the DM + Vit D group did not change the BDNF gene expression in the hippocampus tissue compared to the diabetic control group (DM group) (Table 3). 

## Discussion

Our results showed that after induction of diabetes, FBS, and HbA1C increased, and serum insulin decreased significantly, but glycosylated albumin and serum BDNF did not change significantly. Among factors evaluated in the hippocampus, only phosphorylated pCREB decreased significantly while the gene expression of BDNF and TrkB and total BDNF protein did not change. Administration of vitamin D only improved serum vitamin D and recovered pCREB. HbA1c improved partially, while FBS and insulin did not change significantly; other variables remained non-significant following the intervention in rats. 

Several studies found an association between DM and cognitive impairment, Alzheimer’s disease, and depression (24). The high incidence of cognitive impairment in DM has raised a great deal of concern. Although the underlying mechanism is not fully understood, hippocampal neuroplasticity and neuro-genesis are reported to be sensitive to many pathogenic and stress factors (25). Decreased dendritic branching, hippocampal apoptosis, and reduced hippocampal volume are frequently observed in diabetic patients and diabetic animals (25). Previous studies found that DM-related neurological complications could be due to reduced BDNF concentrations in the CNS (26). BDNF is a major neurotrophic factor (10) involved in neuronal development, synaptogenesis, learning, and memory (9). The actions of BDNF are modulated by its specific receptor, TrkB, which in turn, phosphorylates and activates the transcription factor CREB. pCREB enhances the transcription of many genes, including BDNF itself. Both BDNF and CREB, through the mediation of anti-apoptotic family members, lead to neuronal survival (14)*.* BDNF is mainly produced in the hippocampus and can pass through the brain-blood-barrier (27). The association between BDNF and diabetes has been investigated in experimental and clinical diabetes (19), and low BDNF serum levels have been reported in diabetic humans and mice (19). Furthermore, low BDNF is associated with cognitive impairment in DM patients (28). The expression of CREB and blood levels of BDNF decrease by oxidative stress and overproduction of reactive oxygen species (ROS) and other inflammatory insults (12). Hyperglycemia, AGEs, lipid peroxidation, and chemokines all increase the intracellular oxidant load, which can decrease the BDNF blood level *in situ*. (15). Our results showed that neither BDNF serum level or hippocampal gene expression changed after DM induction. It should be noted that a previous study found that in the RIN5F cell line, only 40 nM of STZ decreased the BDNF expression while STZ at a concentration of 20 nM did not change BDNF gene expression (29); therefore, the exact dose of STZ that can decrease BDNF expression has not been identified yet. In this study, we found that phosphorylated CREB decreased significantly following DM induction. In line with our findings, previous studies reported similar results indicating that STZ induction significantly lowered pCREB (30, 31). Decreased pCREB has been recognized as one of the main reasons for cognitive decline in diabetes. The exact mechanism is not fully understood, but decreased insulin level is suggested as insulin could increase pCREB via MAPK signaling (17, 18). The results of this study showed that the serum level of insulin decreased significantly after T1DM induction. 

Vitamin D deficiency has been identified as one of the main risk factors for dementia. In addition, diabetic neuropathy is reported to be associated with vitamin D deficiency (32, 33). Different antioxidant compounds have been investigated for their possible favorable effects on the BDNF level in diabetes. An interaction between curcumin and dopaminergic receptors, CREB, and phospholipase C is proposed, which can decrease the cortical and cerebellar dysfunction in DM. Therefore, curcumin is suggested as a protective agent against CNS complications in diabetes (6). According to one study, curcumin decreases the oxidative state and restores BDNF levels in DM (34). Other flavonoids like naringenin and tea polyphenols are reported to possess protective effects against oxidative stress-triggered cognitive impairment through up-regulating the protein kinase B(AKT)/CREB/BDNF signaling pathway (12). Administration of vitamin D significantly increased phosphorylated CREB in the hippocampus of T1DM rats. However, BDNF expression, hippocampal TrkB protein, and hippocampal BDNF protein did not change significantly after vitamin D administration. Other *in vitro* studies have reported that phosphorylated CREB increases significantly after treatment with vitamin D in the muscle cell line. The effect of vitamin D supplementation on BDNF protein and BDNF gene expression was inconsistent. Co-administration of vitamin D with a high-fat diet in rats increases the BDNF level in the hippocampus; however, blood levels decreased following the intervention (35, 36). In another *in vivo* study, blood BDNF decreased following aerobic exercise and vitamin D supplementation in ovariectomized rats (37). Treatment of neural stem cells with vitamin D increases BDNF gene expression (38). However, in a Parkinson animal model, calcitriol administration did not change the BDNF level in the hippocampal nuclei (39). In a brain stroke model, vitamin D did not change the BDNF gene expression in brain cortical cells (40). Other *in vitro* studies also showed that despite increased neurotrophin (NT)-4 and NT3 gene expression, BDNF gene expression did not change following vitamin D administration (41). In this study, we investigated the effect of vitamin D administration on the hippocampal CREB-TrkB-BDNF pathway in T1DM rats for the first time. 

VDR and alpha-1-hydroxylase activate vitamin D to its activated form in different areas of the CNS, especially the hippocampus. Vitamin D3 may protect the structure and integrity of neurons through detoxification pathways and neurotrophin synthesis (21). Other pathways, including inducible nitric oxide synthase (iNOS), neurotrophin 3 (NT3), and nerve growth factor (NGF), also have neuroprotective effects (42, 43). Different molecular pathways like MAPK and protein kinase A (PKA) are reported to phosphorylate and activate CREB. Vitamin D acts through binding to VDR. VDR *in situ* results in MAPK activation, which can increase CREB phosphorylation leading to enhanced TrkB and BDNF gene expression (44, 45). 

Our study had some limitations that should be acknowledged. First, the duration of our study was short, which could be the reason why not all factors changed after the intervention. In addition, it seems that investigating the effect of vitamin D administration on post-translation measurements could be helpful, for example, through applying the Western-blot method. Another limitation was the single dose of vitamin, as dose-escalating studies may be more helpful. It seems that another group, i.e., healthy rats receiving vitamin D dissolved in sesame oil, could help find out whether or not the present inconsistency is T1DM specific. Future studies should address these limitations. 

**Table 1:** The sequences of primers used for real-time PCR reactions

Primer		Sequence (5´→ 3´)
TrkB Hippocampus	Forward	5’- TGACGAGTTTGTCCAGGAGA -3’
	Reverse	5’- TTGCTGCTCTCATTGAGGC -3’
BDNF Hippocampus	Forward	5’-GGACATATCCATGACCAGAAAGA-3’
	Reverse	5’-GGCAACAAACCACAACATTATCG-3’
GAPDH	Forward	5’-CATTCTTCCACCTTTGATGCTG-3’
	Reverse	5’-TGGTCCAGGGTTTCTTACTCC-3’

BDNF: Brain-derived neurotrophic factor; TrkB: tropomyosin receptor kinase B (TrkB); GAPDH: glyceraldehyde 3-phosphate dehydrogenase

**Table 2.** Glycemic indices, food intake, body weight, serum vitamin D, and serum BDNF in different experimental groups of Sprague–Dawley rats

Variable	NC	NC+ SO	DM	DM+ Vit D	*P* value
**Initial Weight (g)**	239.4 ± 1.01	241.2 ± 1.64	241.53 ± 2.04	240.14 ± 6.5	0.59
**Final weight (g)**	248.7 ± 1.009	249.6 ± 0.90	215.97 ± 51.70^ a,b^	217 ± 25.29^ a,b^	0.04
**Food intake (g/day)**	25.02 ± 0.02	24.66 ± 1.29	31.17 ± 3.64^a,b^	29.86 ± 1.23 ^a,b^	<0.001
**FBS (mg/dl)**	58.12 ± 8.45	50.62 ± 2.66	349.50 ± 32.25^a,b^	320.62 ± 52.49 ^a,b^	<0.001
**HbA1c (%)**	4.09 ± 0.70	3.92 ± 0.83	8.92 ± 0.64^ a,b^	7.02 ± 0.51^ a,b,c^	<0.001
**Insulin**	2.30 ± 0.50	2.25 ± 0.50	1.16± 0.94^ a,b^	1.01± 0.49^ a,b^	<0.001
**Glycosylated Albumin **	20.24 ± 1.69	18.58 ± 6.94	16.85 ± 4.82	20.03 ± 4.23	0.51
**Serum Vit D (ng/ml)**	28.98 ± 3.44	26.67 ± 4.30	27.60 ± 6.5	40.74 ± 2.16 ^a,b,c^	<0.001
**Serum BDNF**	1.02 ± 0.15	1.11 ± 0.08	0.95 ± 0.34	0.88 ± 0.18	0.18

Results are expressed as mean±SD, one-way ANOVA, and *post hoc* Bonferroni test. DM: diabetes mellitus, FBS: fasting blood sugar, BDNF: brain-derived neurotrophic factor, NC: normal control, SO: sesame oil. a *P*<0.05 compared with the control group, b *P*<0.05 compared with the control group with sesame oil. c *P*<0.05 compared with the diabetic control group

**Table 3:** Hippocampal concentration of phosphorylated CREB and BDNF total protein and gene expression of trkB and BDNF in different experimental groups of rats

**Variable**	**NC**	**NC+ SO**	**DM**	**DM+ Vit D**	*P* **-value**
**Phospho-CREB ** ** (ng/mg) **	24.48 ± 2.39	31.22 ± 5.1	16.79 ± 5.9^b^	39.01± 8.18^a,c^	<0.001
**trkB expression (2 ** ^ΔCt^ **) **	0.052 ± 0.023	0.116 ± 0.119	0.078 ± 0.118	0.042 ± 0.035	0.32
**BDNF expression (2 ** ^ΔCt^ **)**	0.023 ± 0.017	0.014 ± 0.009	0.008 ± 0.007	0.009 ± 0.005	0.28

Results are expressed as mean±SD, one-way ANOVA, and *post hoc* Bonferroni test. DM: diabetes mellitus, NC: normal control, SO: sesame oil; BDNF: brain-derived neurotrophic factor; TrkB: Tropomyosin receptor kinase B; CREB: cAMP-response-element binding protein, a *P*<0.05 compared with the control group, b *P*<0.05 compared with the control group with sesame oil. c *P*<0.05 compared with the diabetic control group

## Conclusion

The results showed that vitamin D restores hippocampal pCREB in diabetic rats while other components of this pathway, including BDNF and TrkB, did not change after the intervention. Although protective agents that affect CNS complications and cognitive impairment in DM are not fully understood, but our findings showed that vitamin D be a promising agent for protection against CNS complications in diabetes.

## References

[1] DeFronzo RA, Ferrannini E, Groop L, Henry RR, Herman WH, Holst JJ (2015). Type 2 diabetes mellitus. Nat Rev Dis Primers.

[2] Control CfD (2017). Prevention. National diabetes statistics report, 2017.

[3] Rathmann W, Giani G (2004). Global Prevalence of Diabetes: Estimates for the Year 2000 and Projections for 2030: Response to Wild et al. Diabetes care.

[4] Chan GC, Tang SC (2015). Diabetic nephropathy: landmark clinical trials and tribulations. Nephrol Dial Transplant..

[5] Leon BM, Maddox TM (2015). Diabetes and cardiovascular disease: Epidemiology, biological mechanisms, treatment recommendations and future research. World J Diabetes.

[6] Kumar TP, Antony S, Gireesh G, George N, Paulose C (2010). Curcumin modulates dopaminergic receptor, CREB and phospholipase C gene expression in the cerebral cortex and cerebellum of streptozotocin induced diabetic rats. J Biomed Sci.

[7] Gonzalez CD, Lee M-S, Marchetti P, Pietropaolo M, Towns R, Vaccaro MI (2011). The emerging role of autophagy in the pathophysiology of diabetes mellitus. Autophagy.

[8] Kitamura T (2013). The role of FOXO1 in β-cell failure and type 2 diabetes mellitus. Nat Rev Endocrinol.

[9] Jiang T, Wang X-q, Ding C, Du X-l (2017). Genistein attenuates isoflurane-induced neurotoxicity and improves impaired spatial learning and memory by regulating cAMP/CREB and BDNF-TrkB-PI3K/Akt signaling. Korean J Physiol Pharmacol.

[10] Janardhanan A, Sadanand A, Vanisree AJ (2016). Nardostachys jatamansi targets BDNF-TrkB to alleviate ketamine-Induced Schizophrenia-Like symptoms in rats. Neuropsychobiology.

[11] Ravichandran V, Kim M, Han S, Cha Y (2018). Stachys sieboldii extract Supplementation Attenuates Memory Deficits by Modulating BDNF-CREB and Its Downstream Molecules, in Animal Models of Memory Impairment. Nutrients.

[12] Qi G, Mi Y, Wang Y, Li R, Huang S, Li X (2017). Neuroprotective action of tea polyphenols on oxidative stress-induced apoptosis through the activation of the TrkB/CREB/BDNF pathway and Keap1/Nrf2 signaling pathway in SH-SY5Y cells and mice brain. Food Funct.

[13] Ye Y-L, Zhong K, Liu D-D, Xu J, Pan B-B, Li X (2017). Huanglian-Jie-du-tang extract ameliorates depression-like behaviors through BDNF-TrkB-CREB pathway in rats with chronic unpredictable stress. Evid Based Complement Alternat Med.

[14] Manji HK, Quiroz JA, Sporn J, Payne JL, Denicoff K, Gray NA (2003). Enhancing neuronal plasticity and cellular resilience to develop novel, improved therapeutics for difficult-to-treat depression. Biol Psychiatry.

[15] Reusch JE, Watson PA, Pugazhenthi S (2006). Disruption of CREB regulated of gene expression in diabetes. Adv Mol Cell Endocrinol.

[16] Dalle S, Quoyer J, Varin E, Costes S (2011). Roles and regulation of the transcription factor CREB in pancreatic beta -cells. Curr Mol Pharmacol.

[17] Begum N, Ragolia L (2000). High glucose and insulin inhibit VSMC MKP-1 expression by blocking iNOS via p38 MAPK activation. Am J Physiol Cell Physiol.

[18] Conejo R, de Alvaro C, Benito M, Cuadrado A, Lorenzo M (2002). Insulin restores differentiation of Ras-transformed C2C12 myoblasts by inducing NF-κB through an AKT/P70S6K/p38-MAPK pathway. Oncogene.

[19] Kalwat MA, Huang Z, McGlynn K, Cobb M (2018). BDNF/TrkB signaling in pancreatic islet beta cells. BioRxiv.

[20] Chiu KC, Chu A, Go VLW, Saad MF (2004). Hypovitaminosis D is associated with insulin resistance and β cell dysfunction. Am J Clin Nutr.

[21] Ahmed HH, El Dayem SMA, Foda FMA, Mohamed HA (2015). Significance of vitamin D in combination with calcium in modulation of depression in the experimental model. Der Pharma Chemica.

[22] Latimer CS, Brewer LD, Searcy JL, Chen K-C, Popović J, Kraner SD (2014). Vitamin D prevents cognitive decline and enhances hippocampal synaptic function in aging rats. Proc Natl Acad Sci U S A.

[23] Schmittgen TD, Livak KJ (2008). Analyzing real-time PCR data by the comparative C T method. Nat Protoc.

[24] Cholerton B, Baker LD, Montine TJ, Craft S (2016). Type 2 diabetes, cognition, and dementia in older adults: toward a precision health approach. Diabetes Spectr.

[25] Ho N, Sommers MS, Lucki I (2013). Effects of diabetes on hippocampal neurogenesis: links to cognition and depression. Neurosci Biobehav Rev.

[26] Nitta A, Murai R, Suzuki N, Ito H, Nomoto H, Katoh G (2002). Diabetic neuropathies in brain are induced by deficiency of BDNF. Neurotoxicol Teratol.

[27] Ho N, Sommers MS, Lucki I (2013). Effects of diabetes on hippocampal neurogenesis: links to cognition and depression. Neurosci Biobehav Rev.

[28] Zhen YF, Zhang J, Liu XY, Fang H, Tian LB, Zhou DH (2013). Low BDNF is associated with cognitive deficits in patients with type 2 diabetes. Psychopharmacology.

[29] Bathina S, Das UN (2018). Dysregulation of PI3K-Akt-mTOR pathway in brain of streptozotocin-induced type 2 diabetes mellitus in Wistar rats. Lipids Health Dis.

[30] Xiang Q, Zhang J, Li C-Y, Wang Y, Zeng M-J, Cai Z-X (2015). Insulin resistance-induced hyperglycemia decreased the activation of Akt/CREB in hippocampus neurons: molecular evidence for mechanism of diabetes-induced cognitive dysfunction. Neuropeptides.

[31] Alvarez-Nölting R, Arnal E, Barcia JM, Miranda M, Romero FJ (2012). Protection by DHA of early hippocampal changes in diabetes: possible role of CREB and NF-κB. Neurochemical research.

[32] Rubin M (2015). vitamin D and Diabetic Neuropathy. Internal Medicine Alert.

[33] McCann JC, Ames BN (2008). Is there convincing biological or behavioral evidence linking vitamin D deficiency to brain dysfunction?. FASEB J.

[34] Franco-Robles E, Campos-Cervantes A, Murillo-Ortiz BO, Segovia J, López-Briones S, Vergara P (2013). Effects of curcumin on brain-derived neurotrophic factor levels and oxidative damage in obesity and diabetes. Appl Physiol Nutr Metab.

[35] Farhangi MA, Mesgari-Abbasi M, Nameni G, Hajiluian G, Shahabi P (2017). The effects of vitamin D administration on brain inflammatory markers in high fat diet induced obese rats. BMC Neurosci.

[36] Hajiluian G, Nameni G, Shahabi P, Mesgari-Abbasi M, Sadigh-Eteghad S, Farhangi MA (2017). Vitamin D administration, cognitive function, BBB permeability and neuroinflammatory factors in high-fat diet-induced obese rats. Int J Obes.

[37] Babaei P, Shirkouhi SG, Hosseini R, Tehrani BS (2017). Vitamin D is associated with metabotropic but not neurotrophic effects of exercise in ovariectomized rats. Diabetol Metab Syndr.

[38] Shirazi HA, Rasouli J, Ciric B, Rostami A, Zhang G-X (2015). 1, 25-Dihydroxyvitamin D3 enhances neural stem cell proliferation and oligodendrocyte differentiation. Exp Mol Pathol.

[39] Smith MP, Fletcher-Turner A, Yurek DM, Cass WA (2006). Calcitriol protection against dopamine loss induced by intracerebroventricular administration of 6-hydroxydopamine. Neurochem Res.

[40] Tetich M, Dziedzicka-Wasylewska M, Kuśmider M, Kutner A, Leśkiewicz M, Jaworska-Feil L (2005). Effects of PRI-2191—a low-calcemic analog of 1, 25-dihydroxyvitamin D3 on the seizure-induced changes in brain gene expression and immune system activity in the rat. Brain Res.

[41] Neveu I, Naveilhan P, Baudet C, Brachet P, Metsis M (1994). 1, 25-dihydroxyvitamin D3 regulates NT-3, NT-4 but not BDNF mRNA in astrocytes. Neuroreport.

[42] Eyles DW, Smith S, Kinobe R, Hewison M, McGrath JJ (2005). Distribution of the vitamin D receptor and 1α-hydroxylase in human brain. J Chem Neuroanat.

[43] Balion C, Griffith LE, Strifler L, Henderson M, Patterson C, Heckman G (2012). Vitamin D, cognition, and dementia: a systematic review and meta-analysis. Neurology.

[44] Tardito D, Perez J, Tiraboschi E, Musazzi L, Racagni G, Popoli M (2006). Signaling pathways regulating gene expression, neuroplasticity, and neurotrophic mechanisms in the action of antidepressants: a critical overview. Pharmacol Rev.

[45] Berdanier CD, Moustaid-Moussa N (2001). Nutrient-gene interactions in health and disease.

[46] Davis E, Keating B, Byrne G, Russell M, Jones T (1998). Impact of improved glycaemic control on rates of hypoglycemia in insulin dependent diabetes mellitus. Arch Dis Child.

